# 1701. Pharmacokinetics in Subjects with Renal Impairment in a Contezolid Complicated Skin and Soft Tissue Infection Phase 3 Clinical Trial

**DOI:** 10.1093/ofid/ofac492.1331

**Published:** 2022-12-15

**Authors:** Edward Fang, Huahui Yang, Hong Yuan

**Affiliations:** MicuRx Pharmaceuticals Inc, San Carlos, California; MicuRx Pharmaceuticals Inc, San Carlos, California; MicuRx Pharmaceuticals Inc, Foster City, CA, USA, shanghai, Shanghai, China

## Abstract

**Background:**

Contezolid (CZD; MRX-I) is a novel oral (PO) oxazolidinone with potent activity against Gram-positive pathogens, including methicillin-resistant *Staphylococcus aureus* (MRSA) and vancomycin-resistant *Enteroccocus* (VRE). In a Phase 3 (Ph3) complicated skin and soft tissue infections (cSSTI) clinical trial (MRX-I-06), 719 subjects were randomized to either CZD 800 mg PO q12h or linezolid (LZD) 600 mg PO q12h with a treatment duration of 7-14 days in both groups. CZD was demonstrated to be noninferior to LZD for the primary efficacy outcome, and overall safety assessments were comparable to LZD. The most common treatment emergent adverse events (TEAEs) in both the CZD and LZD groups were gastrointestinal; however, hematologic laboratory abnormalities and TEAEs were less common with CZD. In June 2021, CZD was approved in China for cSSTI. Sequential therapy with intravenous contezolid acefosamil (CZA; double prodrug of CZD) followed by CZD PO is being evaluated in global Ph3 diabetic foot infection (DFI) and acute bacterial skin and skin structure infection (ABSSSI) clinical trials. Because patients with diabetes commonly have diminished kidney function, pharmacokinetics (PK) in renally impaired subjects were evaluated in MRX-I-06.

**Methods:**

In MRX-I-06, subjects enrolled included those with estimated creatinine clearance (CLcr) of 60 to < 90 mL/min (mild impairment) and 30 to < 60 mL/min (moderate impairment); no dose adjustments were made for renal status. In a subset of the CZD group, PK was evaluated for subjects with no (CLcr ≥90 mL/min), mild, and moderate renal impairment.

**Results:**

The CZD PK subset had 63 subjects, of which 19 had renal impairment (18 mild, 1 moderate). CZD subjects with CLcr above and below 90 mL/min had similar PK values, and CLcr was not a significant covariate (below are means ± SD). There was no indication that the variability in CL/F or in AUC was larger due to renal impairment, and the average AUC and C_max_ were comparable between subjects with renal impairment and those with normal renal function.

**Figure:**
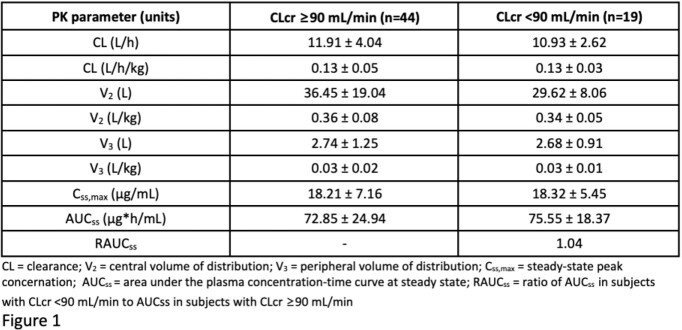

**Conclusion:**

In a Ph3 cSSTI clinical trial, PK in renally impaired subjects who received CZD appeared similar to those without dysfunction, supporting current Ph3 global DFI and ABSSSI studies with CZD and CZA which will enroll subjects with reduced kidney function.

**Disclosures:**

**Edward Fang, MD**, MicuRx Pharmaceuticals Inc: Employee **Huahui Yang, MS**, MicuRx Pharmaceuticals Inc: Employee.

